# Temperature-Responsive Poly(ɛ-caprolactone) Cell Culture Platform with Dynamically Tunable Nano-Roughness and Elasticity for Control of Myoblast Morphology

**DOI:** 10.3390/ijms15011511

**Published:** 2014-01-21

**Authors:** Koichiro Uto, Mitsuhiro Ebara, Takao Aoyagi

**Affiliations:** Biomaterials Unit, International Center for Materials Nanoarchitectonics (WPI-MANA), National Institute for Materials Science (NIMS), 1-1 Namiki, Tsukuba, Ibaraki 305-0044, Japan; E-Mails: uto.koichiro@nims.go.jp (K.U.); ebara.mitsuhiro@nims.go.jp (M.E.)

**Keywords:** temperature-responsive polymers, dynamic cell culture, poly(ɛ-caprolactone), elasticity, nano-roughness

## Abstract

We developed a dynamic cell culture platform with dynamically tunable nano-roughness and elasticity. Temperature-responsive poly(ɛ-caprolactone) (PCL) films were successfully prepared by crosslinking linear and tetra-branched PCL macromonomers. By optimizing the mixing ratios, the crystal-amorphous transition temperature (*T*_m_) of the crosslinked film was adjusted to the biological relevant temperature (~33 °C). While the crosslinked films are relatively stiff (50 MPa) below the *T*_m_, they suddenly become soft (1 MPa) above the *T*_m_. Correspondingly, roughness of the surface was decreased from 63.4–12.4 nm. It is noted that the surface wettability was independent of temperature. To investigate the role of dynamic surface roughness and elasticity on cell adhesion, cells were seeded on PCL films at 32 °C. Interestingly, spread myoblasts on the film became rounded when temperature was suddenly increased to 37 °C, while significant changes in cell morphology were not observed for fibroblasts. These results indicate that cells can sense dynamic changes in the surrounding environment but the sensitivity depends on cell types.

## 1. Introduction

Temporal variations of microenvironments are thought to be important in a wide variety of contexts including development, differentiation, and morphogenesis of cells, as well as progression of diseases and maintenance of homeostasis [1–3]. Recent reports have revealed that the stiffness and topography of the matrix direct cell fate [4–6]. For example, substrate stiffness has been demonstrated to be a key control parameter in the mechanotransduction signaling pathways by up- and down-regulating cell adhesion molecules [7]. Although many studies have shown that the stiffness or topography of the synthetic substrate can influence cell fate, current efforts are centered on rather static effects because properties of synthetic substrates are usually constant in time [3,8,9]. Therefore, much attention has been focused on the designing of dynamic cell culture substrates or matrices with tunable abilities [10,11]. Recent examples of dynamic materials include temperature-responsive polymers such as poly(*N*-isopropylacrylamide) (PNIPAAm). PNIPAAm-grafted surfaces enable reversible hydrophilic/hydrophobic alterations with temperature changes, which allow the cultivation of anchor-dependent mammalian cells at 37 °C, and intact cells or intact cell sheets can be collected after reducing temperature [12,13]. Immobilization of cell adhesive peptides [14] or carbohydrates [15] on the temperature-responsive surfaces also facilitate the selective adhesion and collection of cells. Photo-responsive polymer-based substrates can also regulate cellular functions spatially since the light irradiation can be applied locally with subcellular resolution [16,17]. A photocleavable group, for example, has been utilized to study collective cell migration [17]. Anseth and coworkers have reported a strategy to create photodegradable poly(ethylene glycol)-based hydrogels for remote manipulation of gel properties *in situ*. They successfully demonstrated dynamic morphological control of adhered cells by inducing temporal changes of viscoelastic property upon light irradiation [10,18]. Tanaka and coworkers reported that thin hydrogels based on ABA-type triblock copolymer, which is composed of pH-sensitive poly(2-(diisopropylamino)ethyl methacrylate) as A blocks and biocompatible poly(2-(methacryloyloxy)-ethyl phosphorylcholine) as B blocks, showed reversible modulation of Young’s modulus by 30-fold in a modest pH change from 7.0–8.0. This pH dependent elastic transition also allowed the dynamic modulation of cell-substrate contacts to induce the morphological transition of myoblasts [19]. In spite of a considerable amount of ongoing research, however, the proposed systems not only change the mechano-structural properties of the substrates such as elasticity or topography, but also influence the physico-chemical properties such as surface wettability or swelling ratio. From this regard, we have been developing semi-crystalline poly(ɛ-caprolactone) (PCL) films with different elasticity and topography but similar surface wettability [20,21].

PCL is an important class of the biocompatible and biodegradable synthetic polymers, which has been approved for biomedical applications by the US Food and Drug Administration (FDA). PCL has been widely studied as biodegradable scaffolds in tissue engineering or implantable devices in biomedical fields [22,23]. Since PCL is a semi-crystalline polymer that has a melting temperature (*T*_m_) over which the mobility of polymer chains changes dramatically, it has been also considered as a class of temperature-responsive polymers. One of the advantages of PCL over other temperature-responsive polymers is that surface properties such as wettability and charge are independent of temperature. Therefore, the surface stiffness and roughness can transition without changing cell-surface interactions. We have previously shown how mechanical properties of PCL substrate influence cell behavior using PCL films with a static elastic modulus ranging from 0.9–133 MPa [24–26]. Although the elastic moduli are supra-physiological compared to native tissue, mesenchymal stem cells (MSCs) showed a specific response to substrate stiffness—in terms of adhesion—as a result of differential focal adhesion assembly [25]. In this study, the effects of dynamic changes in the elasticity and surface roughness of substrate on cell behavior were investigated by the induction of the crystal-amorphous transition of PCL. We first prepared PCL films with *T*_m_ in the biological relevant temperature range. Both elasticity and surface roughness were analyzed by a tensile test and atomic force microscope (AFM) at crystalline and amorphous states, respectively. Then, we analyzed time-dependent changes in cell behavior on the PCL films during the crystal-amorphous transition using fibroblasts and myoblasts.

## 2. Results and Discussion

### 2.1. Crystal-Amorphous Transition Temperature

In this study, we synthesized branched PCL by ring-opening polymerization of ɛ-caprolactone from hydroxyl end-group of tetramethylene glycol or pentaerythritol as described in the experimental section. PCL is semi-crystalline aliphatic polyester which has a *T*_m_ of *circa* (ca.) 60 °C. This feature has a great interest in order to shed some light on “on-off” crystal-amorphous transition property especially for the development of dynamic cell culture substrates (Figure S1). In other words, the mechanical property can be easily tuned by changing the surrounding temperature. However, the high *T*_m_ value has limited the potential use of PCL in mild conditions. To overcome this shortcoming, in this study, two-branched (2b) and four-branched (4b) PCL macromonomers were simply mixed in xylene at the concentration of 45 wt % and crosslinked in the presence of BPO as an initiator (10 wt %).

**Figure 1.**
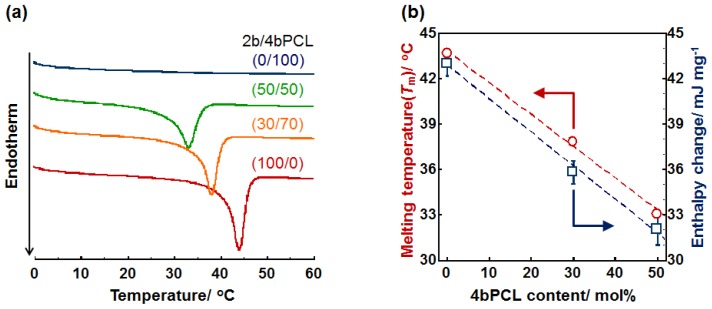
(**a**) DSC curves of crosslinked poly(ɛ-caprolactone) (PCL) films composed of 2b- and 4b-PCL; (**b**) The *T*_m_ and endothermic enthalpy change of crosslinked PCL films as a function of 4b-PCL content.

Figure 1a shows the differential scanning calorimetry (DSC) thermograms of crosslinked PCL films composed of 2b- and 4b-PCLs. The endothermic peaks correspond to the *T*_m_ of the films. This analysis was performed because the *T*_m_ and crystallinity of the films play an important role in affecting the thermally induced mechanical property described in the following section. Crosslinked 4b-PCL (0/100) was completely amorphous at ambient temperature and did not show an endothermic peak. On the other hand, the crosslinked 2b-PCL (100/0) film showed an extremely sharp transition over the *T*_m_ around 44 °C. Interestingly, increasing 4b-PCL content leads to a near linear decrease in *T*_m_ and endothermic enthalpy change (Δ*H*) (Figure 1b). This implies that an increase in crosslinking density reduces the crystallization of freely mobile polymer chains. By optimizing the mixing ratios, therefore, the *T*_m_ of the crosslinked film can be adjusted to the biological relevant temperature. For example, the film with 50/50 wt % of 2b/4b-PCL had a *T*_m_ around 33 °C. The ability to tune the switching temperature close to the biological temperature allows for cell culture studies of these films.

### 2.2. Elastic Modulus

**Figure 2.**
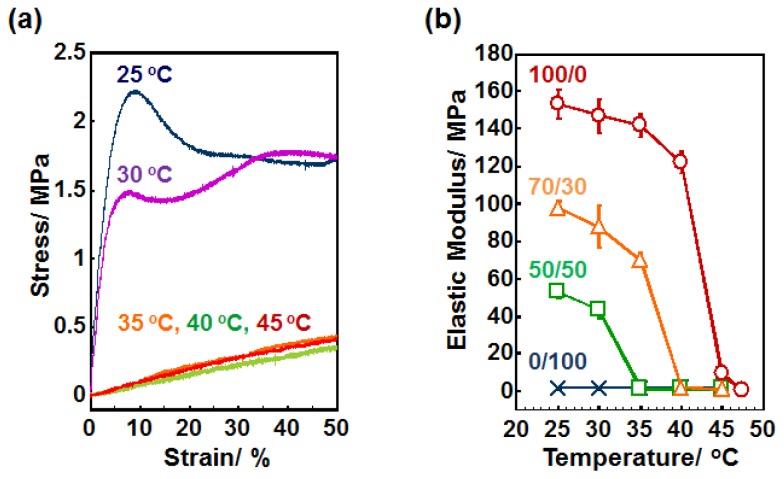
(**a**) Stress-strain curves of crosslinked PCL films with 50/50 of 2b/4b ratio at 25, 30, 35, 40 and 45 °C; (**b**) Elastic modulus of crosslinked PCL films with various 2b/4b ratios as a function of temperature.

Next, we examined the mechanical property of the crosslinked PCL films at different temperatures by a tensile test. The representative stress-strain curves of the film (2b/4b = 50/50) are shown in Figure 2a. The films displayed distinct yield strengths at 2.2 and 1.5 MPa at 25 and 30 °C, respectively. On the other hand, the crosslinked films show significantly lower values at 35 °C or above. This is because the PCL films became amorphous above the *T*_m_ (around 33 °C), resulting in less deformation resistance and in becoming more rubbery. Similar observations were obtained for other PCL films with different compositions (Figure S2 in the supplementary information). To investigate the softening transition over the *T*_m_, the Young’s modulus was plotted against temperature (Figure 2b). Young’s modulus was determined from the initial slope of stress-strain curve at each temperature. The 4b-PCL with 0/100 of 2b/4b, which did not show an endothermic peak in DSC measurement, showed significantly lower elastic moduli (~1.5 MPa) and those values were independent of temperature in the range 25–45 °C. For the 50/50, 70/30, and 100/0 samples, on the other hand, the moduli decrease gradually with temperature until a rapid softening transition occurs between 30 and 35 °C, 35 and 40 °C, and 40 and 45 °C, respectively. The transition temperature ranges are well consistent with the endothermic peak ranges observed in Figure 1a. Importantly, the transition occurred over a narrow temperature range (~5 °C) and were associated with a large elastic modulus decreases from 42.2, 68.9, and 112.7 MPa for 50/50, 70/30, and 100/0 samples, respectively.

### 2.3. Surface Morphology and Wettability

**Figure 3.**
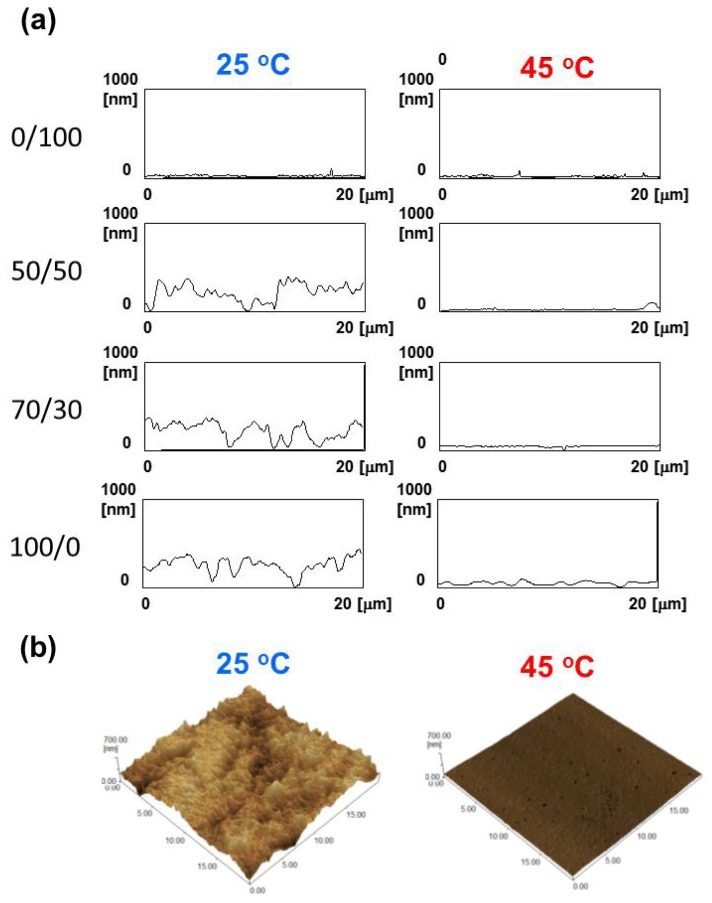
(**a**) Topographic surface images of PCL films observed by AFM at 25 °C (left) and 45 °C (right); (**b**) 3D AFM images of 70/30 PCL film observed at 25 °C (left) and 45 °C (right). All images were obtained in the 20 μm × 20 μm scan range.

In addition to bulk properties, surface properties such as morphology and wettability are important for cell adhesion and spreading process [27,28]. The morphologies of the PCL surfaces were imaged by AFM at 25 and 45 °C. Although temperature can limit the sensitivity and resolution of the AFM measurement, this is beyond the scope of this article and is not reported here. Figure 3 shows topographic surface images of PCL films at 25 °C and 45 °C observed by AFM over an area of 20 × 20 μm^2^. In amorphous PCL (0/100), the large difference was not observed between 25 and 45 °C. The surface mean roughness values (*R*_a_) were 6.2 and 6.1 nm, respectively. For the 50/50, 70/30, and 100/0 PCL films which have the *T*_m_ between 25 and 45 °C, on the other hand, irregular rough structures were observed at 25 °C due to the crystallization of PCL (left images in Figure 3a). The *R*_a_ values for 50/50, 70/30 and 100/0 PCL films were found to be 63.4, 83.6, and 85.5 nm, respectively. It is well-known that morphology or patterns of crystalline structures of PCL can be influenced by the growth kinetics, temperature, composition, *etc.* [29]. In this study, films with higher *T*_m_ showed larger crystals at 25 °C. When the films were heated at 45 °C and imaged again, the surfaces became relatively smooth with the *R*_a_ values of 12.4, 13.4, and 27.6 nm for 50/50, 70/30, and 100/0, respectively (right images in Figure 3a). Figure 3b shows the representative 3D AFM images of 70/30 PCL films at 25 (left) and 45 °C (right). Clear difference can be also seen in the phase contrast AFM images between two surfaces as shown in the supporting information (Figure S3). In general, the AFM signal is influenced not only by the topography but also by e.g., local elasticity variations or changes in the interaction potential if the surface is made up of different materials [30]. The phase shift can be thought of as a delay in the oscillation of the cantilever as is moves up and down in and out of contact with the sample. Therefore, phase contrast is one of the most commonly used techniques for mechanical or viscoelastic characterization of sample surfaces. The phase images of 70/30 PCL films measured at 25 and 45 °C are found to be darker and brighter corresponding to less and more stiff, respectively. These results indicate that not only bulk stiffness but also surface stiffness of PCL film have been dramatically changed by heating.

**Figure 4.**
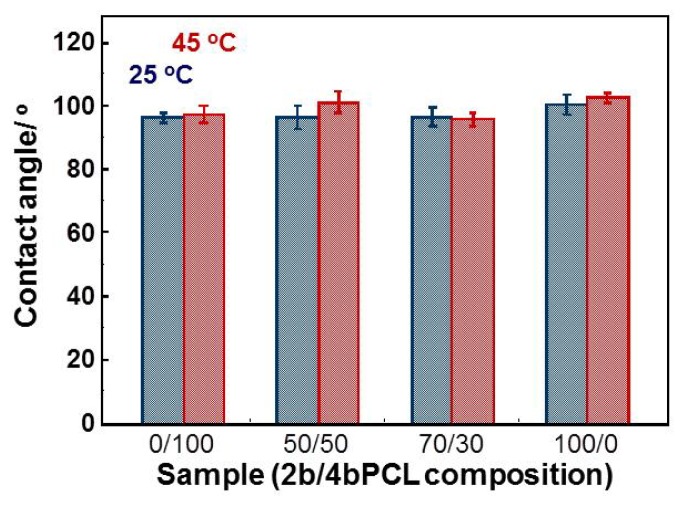
Contact angles on PCL films at 25 and 45 °C. Error bars represent standard deviation for *n* ≥ 6 measurements of independent experiments.

Since the surface wettability plays important role for protein adsorption and following the cell adhesion process, we also carried out the contact angle measurements of crosslinked PCL films at 25 and 45 °C (Figure 4). Interestingly, all samples showed the similar surface wettability (around 95.8°–102.5°) regardless of the temperature tested. This is one of the greatest advantages of PCL over other temperature-responsive polymers, surface wettability of which are dramatically influenced by temperature. Table 1 summarizes the characteristics of PCL films at 25 and 45 °C. The *T*_m_ of the crosslinked PCL films proportionally decreased with increasing 4b content because an increase in crosslinking density hinders crystallization of PCL. In particular, the film with 50/50 wt % mixing ratio of 2b/4b had a *T*_m_ around 33 °C. The crystal-amorphous transition occurred over a few degrees and was associated with large decreases in both elastic modulus and surface roughness from 52.9–1.1 MPa and from 63.4–12.4 nm, respectively. The surface contact angle around 100° was independent of temperature. Therefore, we used 50/50 PCL films for the subsequent cell culture experiments.

### 2.4. Cell Behavior

**Figure 5.**
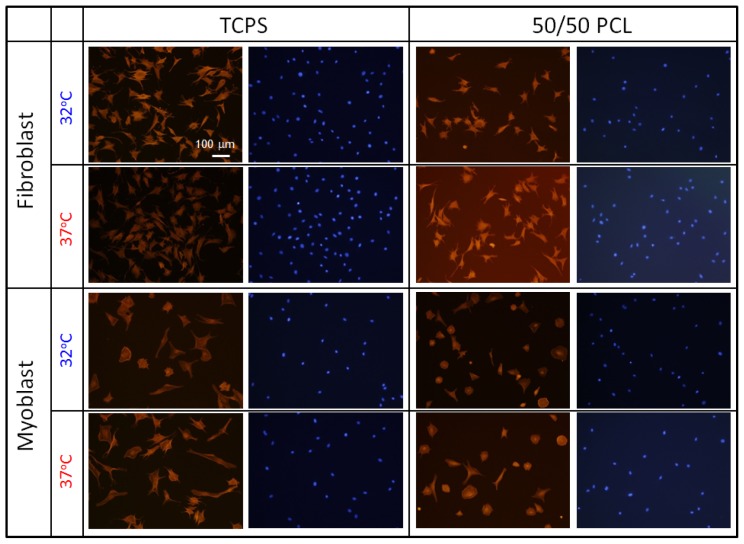
Fluorescence microscopy images of fibroblasts and myoblasts seeded on TCPS (left) and 50/50 PCL (right). Cells were cultured at 32 or 37 °C for 24 h. The cells were fixed with paraformaldehyde and treated with Rhodamine phalloidin for F-actin staining (red) and DAPI for nucleus staining (blue).

**Figure 6.**
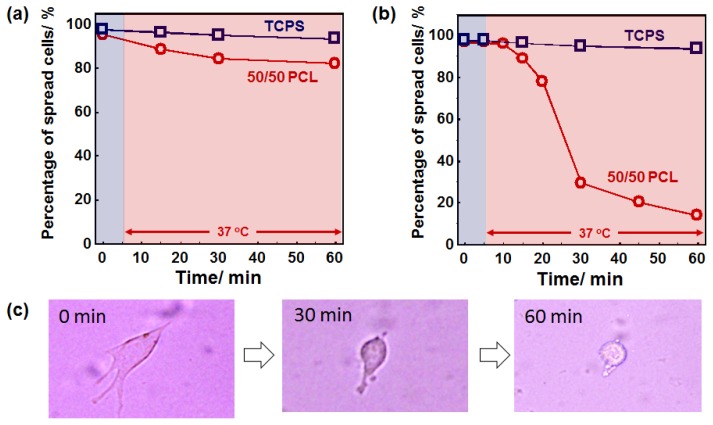
Time-dependent changes in the cell morphologies on PCL films after the crystal-amorphous transition. Cells were cultured on the films at 32 °C for 24 h. The cells were then subjected to a 37 °C heat treatment. The percentage of spread cell numbers on the surface was plotted against time. (**a**) Fibroblast; (**b**) Myoblast; (**c**) Phase contrast images of myoblasts on 50/50 PCL film after heat treatment.

As described above, the crosslinked PCL with 50/50 of 2b/4b ratio can induce dramatic changes in both elasticity and surface roughness over 33 °C without changing their surface wettability (Figure S4 and Table S1). To investigate the role of dynamic changes in surface elasticity and roughness on cell adhesion and spreading, cell morphology on the PCL films was observed during the crystal-amorphous transition. Since the response of cells to substrate elasticity and surface roughness has been demonstrated to be highly cell specific, we cultured several types of cells on the PCL films. First, NIH 3T3 fibroblasts and rat skeletal (MYB01) myoblasts were seeded on the 50/50 PCL films and tissue culture polystyrene (TCPS) dishes and cultured for 24 h at static temperature of 32 or 37 °C. Both fibroblasts and myoblasts adhered and spread well irrespective of the substrate. Interestingly, significant differences in the cell morphology were not observed between 32 and 37 °C as seen in Figure 5. Although more myoblasts exhibited a typical myoblast-like morphology when cultured at 37 °C than at 32 °C, this is not due to either surface elasticity or roughness because similar trend can be also observed for TCPS. These results indicate that significant effects of surface elasticity and roughness on cell behavior were not observed when cells were cultured at static condition. Next, cells were subjected to a heat treatment to investigate the effects of dynamic changes in surface elasticity and roughness on cell behavior. Cells were seeded on the films and cultured at 32 °C (below the *T*_m_) for 24 h. Then, the cells were placed on a 37 °C heater (above the *T*_m_) and the cell morphology was continuously monitored. Figure 6 shows time-dependent changes of spreading cell percentages for (a) fibroblasts and (b) myoblasts. Neither cell morphological changes nor cell detachment occurred on TCPS surfaces over the 60 min imaging time. This indicates that temperature change from 32 and 37 °C does not affect cell morphology in this study. The myoblasts spread on the 50/50 PCL films, however, dramatically changed their shape upon heating. Immediately after heating, myoblasts lost their flattened morphology and became rounded. More than 70% of cells changed their morphology to rounded shapes within 30 min (Figure 6c). The rounded cells finally come off the surface when mildly agitated. Fibroblasts also responded in a similar manner, but only 20% of cells became rounded within the same period.

**Figure 7.**
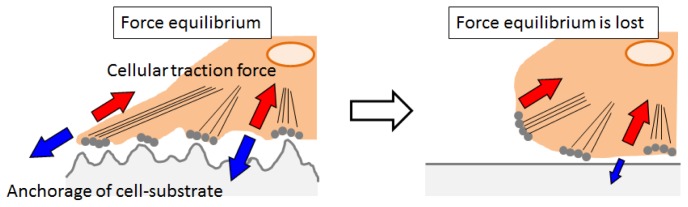
The possible mechanisms of cell detachment in response to dynamic changes in surface crystallinity. There is equilibrium between the pulling forces developed by the cytoskeletal dynamics and tensile stress of the substrate. Sudden transition of the surface from crystal to amorphous abolishes tight anchorage of cell-substrate. As a result, the force equilibrium is lost and the remaining tensile forces developed by the cytoskeleton cause cell rounding.

The exact mechanism of the observed morphological change of myoblasts is of course highly complex and not entirely understood at present. However, we can speculate on the possible mechanisms that give rise to this behavior. Myoblasts are known to undergo reorientation, alignment and remodeling of the cytoskeleton when they experience mechanical stretch and compression in muscle tissue. It has been reported that myoblasts are able to maintain a significant amount of adhesion and contract with the microenvironment during large scale cytoskeletal depolymerization [31]. This result suggests that myoblasts generate a significant amount of traction. Figure 7 shows the possible mechanisms of cell detachment in response to dynamic changes in surface elasticity and roughness. Adhesive cells generate centripetal traction forces mediated by stress fibers on culture surfaces. There is equilibrium between the pulling forces developed by the cytoskeletal dynamics and tensile stress of the substrate [32,33]. Sudden transition of the crystalline surface abolishes tight anchorage of cell-substrate. As a result, the force equilibrium is lost and the remaining tensile forces developed by the cytoskeleton cause cell rounding. Although many studies have demonstrated that myoblasts are sensitive to differences in substrate elastic modulus, those studies used much softer hydrogels than our PCL, which were similar to the elasticity of muscle tissue [19,34,35]. Indeed, the elastic moduli of our PCL are supra-physiological compared to native tissue. But, another report also suggested that myoblasts were insensitive to nanomechanical stimulation on softer substrates while they responded rapidly to a nanomechanical force in a stiffer microenvironment [36]. Taken together, sudden loss of an equilibrium balance between the cell-substrate interfaces would account for the observations in this study. However, future studies are clearly required in order to provide a full and complete mechanism.

## 3. Experimental Section

### 3.1. Fabrication of Crosslinked PCL Films

The poly(ɛ-caprolactone) (PCL) films were prepared by crosslinking tetra-branched PCL with acrylate end-groups in the presence of linear PCL telechelic diacrylates according to a previously reported protocol [20,37,38]. Briefly, linear and tetra-branched PCL were synthesized by ring-opening polymerization of ɛ-caprolactone (CL; Tokyo Chemical Industry (TCI) Co., Ltd, Tokyo, Japan) that was initiated with tetramethylene glycol (Wako Pure Chemical Industries, Ltd, Osaka, Japan) and pentaerythritol (TCI Co., Ltd.) as initiators, respectively. Then, acryloyl chloride (TCI Co., Ltd.) was reacted to the hydroxyl end group of the branched chains. The structures and the molecular weights were estimated by ^1^H NMR spectroscopy (JEOL, Tokyo, Japan) and gel permeation chromatography (JASCO International, Tokyo, Japan), respectively. The average degrees of polymerization of each branch for linear and tetra-branch PCL were 18 and 10, respectively. The equimolar amounts of PCL macromonomers were then dissolved at 45 wt % in xylene containing 2-fold molar excess benzoyl peroxide (BPO; Sigma-Aldrich, St. Louis, MO, USA) to the end-group of macromonomers. The solution was injected between glass slides with a 0.2 mm thick Teflon spacer. Then, thermal polymerization was carried out at 80 °C for 180 min to obtain the cross-linked PCL films. The thermal properties of the branched PCLs were measured by differential scanning calorimetry (DSC, 6100, SEIKO Instruments, Chiba, Japan) at 5 °C/min of programming rate.

### 3.2. Thermo-Mechanical Properties

The mechanical property of crosslinked PCL under the controlled temperature was carried out by thermomechanical experiments [22]. A tensile tester (EZ-S 500N, Shimadzu, Kyoto, Japan) equipped with a thermo chamber (Chromato chamber M-600FN, TAITEC, Saitama, Japan) was used to allow simultaneous tensile test and thermal programs. First, the crosslinked PCL films were heated and equilibrated at measurement temperature ranging from 25 to 45 °C for 1 h. The tensile test was then performed at elongation speed of 5 mm/min to obtain the strain-stress curve at each temperature. The elastic modulus of crosslinked PCLs was calculated from the initial slope of the obtained stress-strain curves.

### 3.3. Topographical Observation by Atomic Force Microscope (AFM)

The surfaces morphology of crosslinked PCL films were observed by atomic force microscopy (AFM) (SPM-9500J3, Shimadzu Co., Kyoto, Japan) with non-contact mode using Si_3_N_4_ cantilever (spring constant; 42 N/m, Nano World, Neuchâtel, Switzerland), and the sample temperature was controlled using a thermo controller. The crosslinked PCL films were heated and equilibrated at 25 °C and 45 °C for 1 h, and AFM measurement was performed to obtain the height and phase images. The surface roughness (*R*_a_) at 25 and 45 °C were estimated from AFM scans on 20 × 20 μm^2^ area.

### 3.4. Contact Angles

The surface wettability of crosslinked PCL at 25 and 45 °C was measured by contact angle measurements (DSA100; KRUSS, Hamburg, Germany). First, the crosslinked PCL films were heated and equilibrated for 1 h, and the contact angle measurement was then conducted in humidity of 70%. Data are averaged from at least three separate experiments for each film and shown with their standard deviation.

### 3.5. Cell Culture

Before cell culture, PCL films were sterilized by low pressure hydrogen peroxide gas plasma system CH-160C (Toho Seisakusho, Tokyo, Japan). The films were pre-incubated in Dulbecco’s modified Eagle’s medium (DMEM; Sigma-Aldrich, St. Louis, MO, USA) in the presence of 10% fetal bovine serum (FBS; Equitech-Bio Inc., Kerrville, TX, USA) and 1% antibiotic-antimycotic (anti-anti; Gibco, Grand Island, NY, USA) and equilibrated in a 32 °C incubator for 1 h. NIH 3T3 fibroblasts and primary myoblasts extracted from rat skeletal muscle (MYB01; F-12, Primary Cell CO., Ltd, Hokkaido University, Sapporo, Japan) were then seeded at a density of 2.0 × 10^4^ cells cm^−2^ on the crosslinked PCL and cultured in DMEM containing 10% FBS and 1% anti-anti at 32 °C. For dynamic cell culture experiments, the cells were transferred to a 37 °C incubator after 24 h of incubation at 32 °C. The cells were subjected to a 37 °C heat treatment for 1 h. The cell morphology before and after heating was continuously monitored and imaged using a phase contrast microscope (Olympus IX71, Tokyo, Japan).

## 4. Conclusions

Temperature-responsive crosslinked PCL with dynamically tunable nano-roughness and elasticity were successfully prepared by crosslinking 2b- and 4b-PCL macromonomers. The crystal-amorphous transition temperature (*T*_m_) of the crosslinked PCL films proportionally decreased with decreasing 4b content. By optimizing the mixing ratios, the *T*_m_ was successfully adjusted to the biological relevant temperature (around 33 °C). The crystal-amorphous transition over the *T*_m_ was associated with large decreases in both elastic modulus and surface roughness, while surface wettability was independent of temperature. Significant effects of the crystal-amorphous transition of PCL films on cell behavior were not observed when cells were cultured at static temperature at both below and above the *T*_m_. However, spread myoblasts on the film became rounded when temperature was suddenly increased to 37 °C. These results indicate that cells are more sensitive to dynamic changes in the surrounding environment than in static condition, but the sensitivity depends on cell types. We believe that the versatility and biologically-friendly nature of PCL materials could potentially enable the realization of novel and diverse applications, especially biomaterial development and basic cell biology.

## Supplementary Information



## Figures and Tables

**Table 1.** Summary of the characterizations of PCL films at different temperatures.

2b/4bPCL	*T*_m_ (°C)	Elasticity (MPa)		Roughness (nm)		Contact angle (°)	

		25 °C	45 °C	25 °C	45 °C	25 °C	45 °C
0/100	-	1.4 (±0.4)	1.5 (±0.1)	6.2	6.1	96.5 (±1.6)	97.4 (±2.4)
50/50	33.0 (±0.1)	52.9 (±3.0)	1.1 (±0.1)	63.4	12.4	96.3 (±3.7)	100.9 (±3.3)
70/30	37.8 (±0.1)	97.6 (±3.6)	0.9 (±0.1)	83.6	13.4	95.8 (±2.1)	96.6 (±2.8)
100/0	43.7 (±0.3)	153.4 (±8.0)	9.3 (±2.7)	85.5	27.6	100.5 (±3.1)	102.5 (±1.5)
