# Supplementary material for: Temperature-Responsive Poly(ɛ-caprolactone) Cell Culture Platform with Dynamically Tunable Nano-Roughness and Elasticity for Control of Myoblast Morphology
Source: Int J Mol Sci. 2014 Jan 21;15(1):1511–24. doi: 10.3390/ijms15011511 (PMC3907883; doi:10.3390/ijms15011511)
Supplement: Supplementary file 1 [file ijms-15-01511-s001.pdf]

Supplementary Information

Figure S1. Digital photographs and topographic surface images of crosslinked PCL with reversibly tunable (**a**) elasticity and (**b**) surface nano-roughness. AFM images at various temperatures were obtained in the 50 µm × 50 µm scan range.

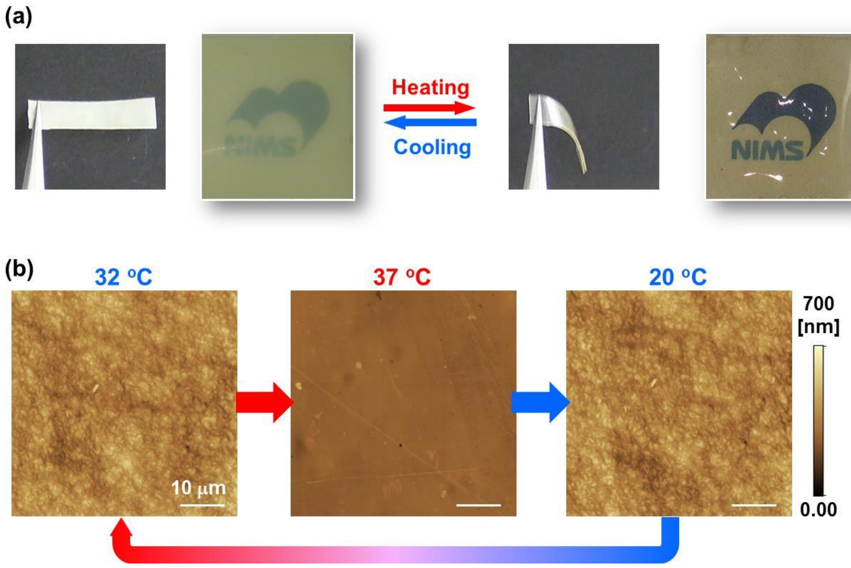

Figure S2. Stress-strain curves of crosslinked PCL with various 2b/4bPCL-m ratios at 25, 30, 35, 40 and 45 ℃.

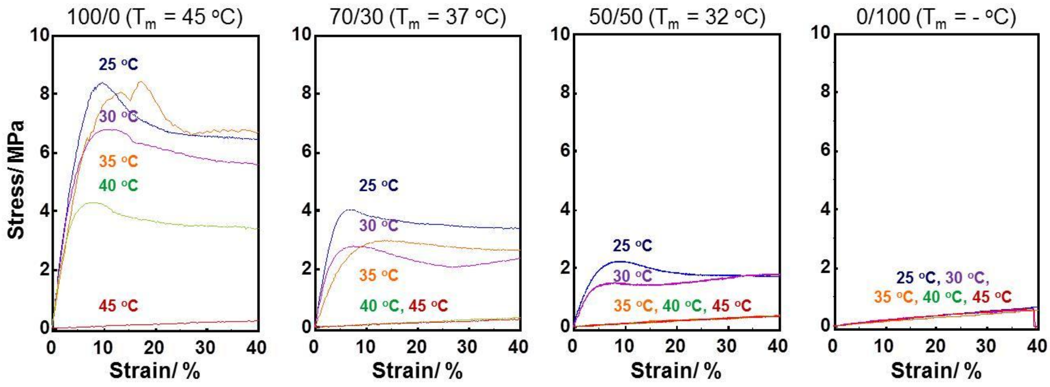

Figure S3. Phase images of PLC films observed by AFM at 25 ℃ (**left**) and 45 ℃ (**right**). All images were obtained in the 20 µm × 20 µm scan range.

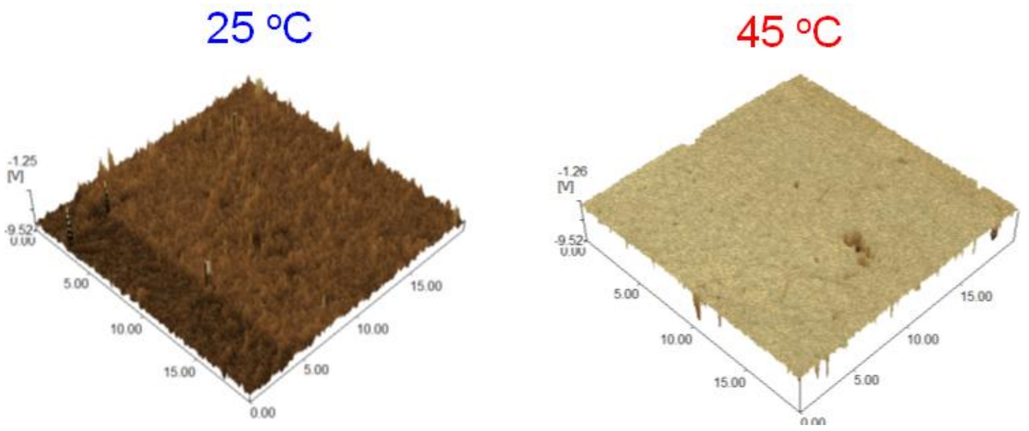

Figure S4. (**a**) Topographic surface and (**b**) 3D AFM images of 50/50 PCL films observed by AFM at 32 °C (left) and 37 °C (right). All images were obtained in the 20 μm × 20 μm scan range.

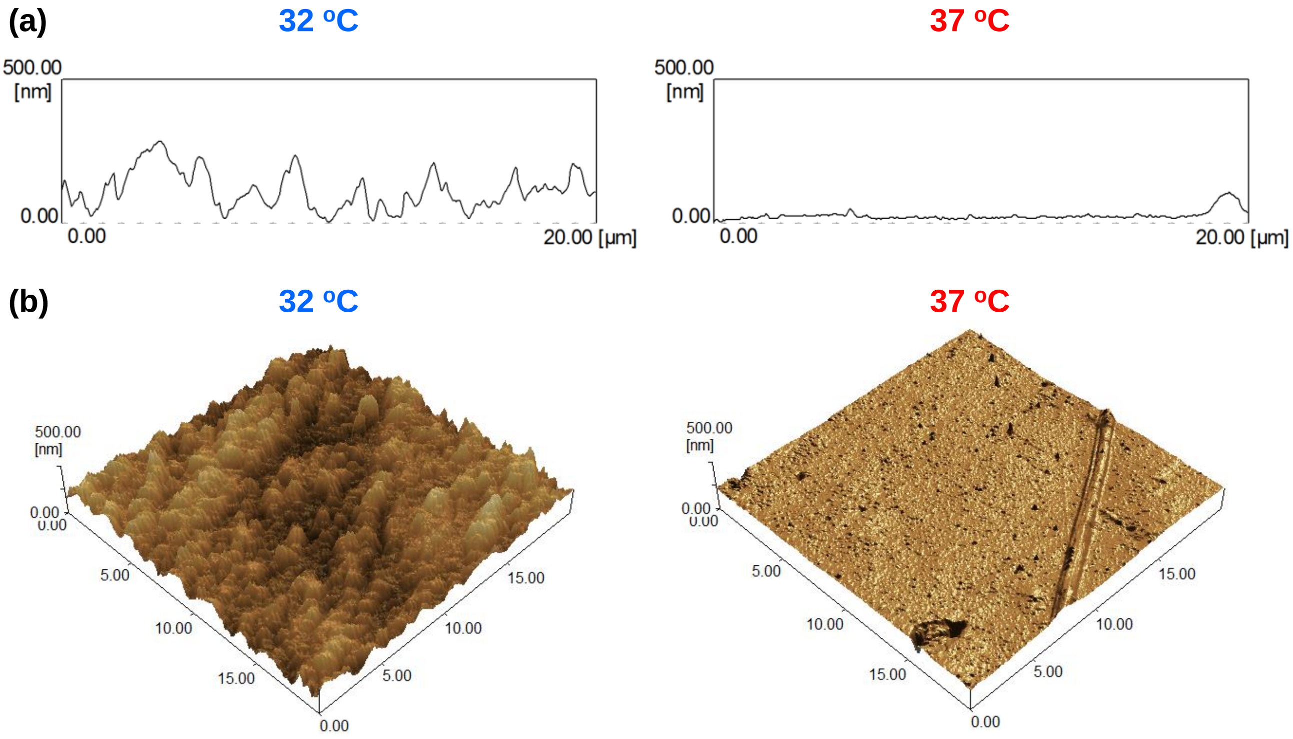

Table S1. Summary of the physical property (elasticity and surface roughness) of 50/50 PCL films in cell culture conditions (32 and 37 °C).

**2b/4bPCL**	**Elasticity (MPa)**		**Roughness (nm)**	
	32 °C	37 °C	32 °C	37 °C
50/50	26.4 (±2.7)	1.1 (±0.3)	63.4	12.4

© 2014 by the authors; licensee MDPI, Basel, Switzerland. This article is an open access article distributed under the terms and conditions of the Creative Commons Attribution license (http://creativecommons.org/licenses/by/3.0/).
